# Trend analysis of *Trichinella* in a red fox population from a low endemic area using a validated artificial digestion and sequential sieving technique

**DOI:** 10.1186/s13567-014-0120-9

**Published:** 2014-11-28

**Authors:** Frits Franssen, Gunita Deksne, Zanda Esíte, Arie Havelaar, Arno Swart, Joke van der Giessen

**Affiliations:** National Institute for Public Health and the Environment, Centre for Zoonoses and Environmental Microbiology, Bilthoven, The Netherlands; Institute of Food Safety, Animal Health and Environment “BIOR”, Riga, Latvia; Division Veterinary Public Health, Institute for Risk Assessment Sciences, Utrecht University, Utrecht, The Netherlands

## Abstract

Freezing of fox carcasses to minimize professional hazard of infection with *Echinococcus multilocularis* is recommended in endemic areas, but this could influence the detection of *Trichinella* larvae in the same host species. A method based on artificial digestion of frozen fox muscle, combined with larva isolation by a sequential sieving method (SSM), was validated using naturally infected foxes from Latvia. The validated SSM was used to detect dead *Trichinella* muscle larvae (ML) in frozen muscle samples of 369 red foxes from the Netherlands, of which one fox was positive (0.067 larvae per gram). This result was compared with historical *Trichinella* findings in Dutch red foxes. Molecular analysis using 5S PCR showed that both *T. britovi* and *T. nativa* were present in the Latvian foxes, without mixed infections. Of 96 non-frozen *T. britovi* ML, 94% was successfully sequenced, whereas this was the case for only 8.3% of 72 frozen *T. britovi* ML. The single *Trichinella* sp. larva that was recovered from the positive Dutch fox did not yield PCR product, probably due to severe freeze-damage. In conclusion, the SSM presented in this study is a fast and effective method to detect dead *Trichinella* larvae in frozen meat. We showed that the *Trichinella* prevalence in Dutch red fox was 0.27% (95% CI 0.065-1.5%), in contrast to 3.9% in the same study area fifteen years ago. Moreover, this study demonstrated that the efficacy of 5S PCR for identification of *Trichinella britovi* single larvae from frozen meat is not more than 8.3%.

## Introduction

*Trichinella* species infect a wide range of mammals, including humans [1,2]. In the European Union, the magnetic stirrer method (EU reference method, EU-RM) according to European regulation EC 2075/2005 [3] is used for individual carcass control of *Trichinella* susceptible animals intended for human consumption and for surveillance of *Trichinella* infections in wildlife. This method includes two consecutive sedimentation steps to isolate *Trichinella* muscle larvae (ML) and has been validated for the detection of live larvae, for which critical control points are well described [4]. To analyse *Trichinella* in wildlife, some adjustments to the magnetic stirrer method are necessary to improve efficiency, like prolongation of digestion time, since meat of wildlife is more difficult to digest. In Europe, the red fox is considered an indicator species for *Trichinella* infections in wildlife and many studies are being carried out to determine the prevalence and infection rate of *Trichinella* in red fox populations [5-13]. Since in Europe the red fox is also a final host for *Echinococcus multilocularis*, a zoonotic parasite and causative agent of alveolar echinococcosis in humans, fox carcasses are deep frozen at −80 °C for minimally one week, to inactivate the infective stage of this fox tapeworm prior to post mortem examination, according to WHO biosafety instructions [14]. Already between −18 and −30 °C, freezing kills *Trichinella* ML within one week [15-18], thereby altering their sedimentation characteristics [4], which is a key factor in the analysis with EU-RM. Gamble [19] showed that live larvae settled with a sedimentation speed of about 2 cm/min in meat digest at 40 °C. This is enough to pass through 2 litres of meat digest in a separatory funnel within 16 min. At 4 °C the sedimentation speed was less, which would prolong the sedimentation time to 24 – 28 min. In contrast, Dyer and Evje [20] recovered only 80% of spiked dead *Trichinella* ML in 2 litres *Trichinella*-free meat digest after one hour of sedimentation (twice the time routinely used in EU-RM).

Well before the EU-RM was established, Henriksen [21] successfully used a filtration method to isolate dead *Trichinella* ML from experimentally infected rabbits. Enemark et al. [9] used 22 μm disposable filters to retain ML after artificial digestion of fox fore legs that had been kept at −20 °C for three to ten months prior to analysis. Retained ML were visualized by subsequent iodine/hypochlorite staining, which renders these larvae unsuitable for molecular species identification.

Van der Giessen et al. [8] used the Trichomatic^35^ method, an automated system by which naked *Trichinella* larvae were isolated on a 14 μm mesh size nylon filter for subsequent microscopical examination. Isolated individual larvae were identified as *Trichinella britovi*, using a single larva PCR and reversed line blot analysis as described by Rombout et al. [22].

In this study, we describe validation of an artificial digestion method using the magnetic stirrer method, followed by a sequential sieving step to isolate dead *Trichinella* larvae from naturally infected fox muscle samples. We show that the recovery rate of spiked dead *Trichinella* larvae in meat digest is 60% using EU-RM, while the recovery rate using SSM is 92%, making SSM the technique of choice to detect dead *Trichinella* larvae in frozen meat. Consequently, the most sensitive technique was used to analyse the recovery rate of *Trichinella* larvae before (EU-RM, live larvae) and after (SSM, dead larvae) freezing of naturally infected fox samples. Moreover, the efficacy of molecular identification was studied on isolated ML originating from foxes from an endemic area, before and after freezing. The validated sequential sieving method was used to study *Trichinella* prevalence in the red fox population in the eastern border region of the Netherlands. Obtained *Trichinella* prevalence was compared to historical data to analyse trends in time.

## Materials and methods

### Animals and *Trichinella* larvae

The left Foreleg of 35 *Trichinella* positive (EU-RM) [3] non-frozen red foxes from Latvia were collected during routine inspection at the Institute of Food Safety, Animal Health and Environment BIOR (Riga, Latvia). These animals originated from all four Latvian regions (Vidzeme 6, Zemgale 7, Latgale 9 and Kurzeme 11 individuals, 2 not specified). After primary analysis of the muscle samples by EU-RM without freezing, the forelegs were frozen and kept at −80 °C for one to two weeks, after which a second muscle sample from the same foreleg was tested with SSM at BIOR. A digestion time of 30-40 min was used for artificial digestion as described [3,4]. After detection of ML, isolated *Trichinella* larvae were kept in 96% ethanol at room temperature until further use. For analysis with multiplex PCR [23], pools of five *Trichinella* ML were isolated from 30 foxes from all four regions of Latvia (Vidzeme 6, Zemgale 7, Latgale 8 and Kurzeme 9 individuals). For single larva PCR, individual *Trichinella* ML from the same 30 foxes that were found positive both before and after freezing, were transferred to 5 μL of DNAse free water and stored at −20 °C until further use.

Live *Trichinella britovi* larvae for the validation of detection by sequential sieving were obtained from a farmed wild boar, which tested positive during regular meat inspection in Latvia (Zemgale region, Latvia, Institute of Food Safety, Animal Health and Environment BIOR).

*Trichinella spiralis* (ISS 14) larvae for use in spike experiments were obtained from experimentally infected mice by the EU-RM. This work was approved by the Ethical Committee of the Dutch National Institute for Public Health and the Environment (RIVM) (DEC permit number 201200223).

**Figure 1:**
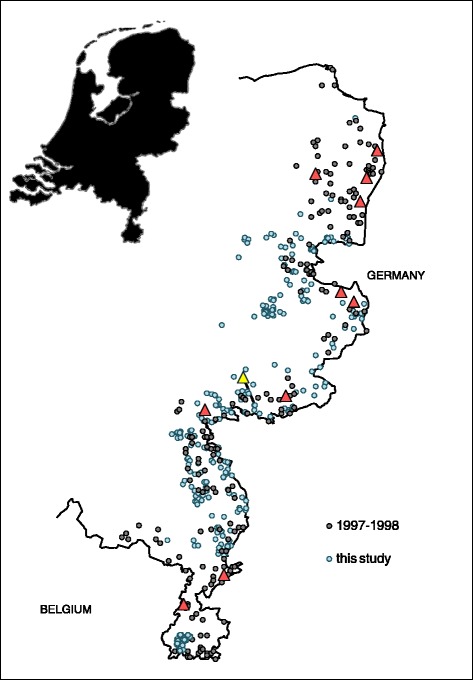
**Geographical origin of Dutch red foxes.** At the eastern border of the Netherlands (outline) 369 foxes were collected during the period 2010 -2013 (blue circles), of which one fox was positive for *Trichinella* (yellow triangle). In contrast, in a similar study in 1997-1998 (grey circles), eleven *Trichinella* positive foxes (red triangles) were found in a collection sample of 276 red foxes, ten of which in the same study area [21].

For Trichinella survey in the Netherlands from October 2010 - April 2013, 369 Dutch foxes were collected by hunters from the border region with Germany in the east and Belgium in the south (Figure 1). The majority of foxes (287) was collected during the hunting season November 2010 - April 2011. Collected foxes were sent to RIVM, Bilthoven, the Netherlands. Upon arrival, fox carcasses were stored at −80 °C to inactivate the eggs of possibly present *E. multilocularis* [24] according to WHO guidelines [14]. After a minimum period of one week, carcasses were thawed at approximately 10 °C and dissected. Muscles of both lower forelegs of each fox were collected and 15 g of muscle tissue was analysed for *Trichinella*, using the validated SSM.

### Validation experiments

Crucial steps of the EU-RM for the detection of Trichinella larvae are complete digestion of muscle tissue and high effectivity of the procedure to isolate *Trichinella* ML. To validate the method for detection of dead *Trichinella* larvae in frozen meat samples, the process was separated into three stages.

1. Isolation and detection of dead larvae. The efficacy of dead *Trichinella* ML isolation using EU-RM and SSM was compared by spiking dead Trichinella ML in meat digest and subsequent recovery of ML. The sequential sieving method to detect *Trichinella* larvae was further validated by adding live or dead larvae to water or *Trichinella*-free meat digest and recovery by SSM.

2. Feasibility of the use of *Trichinella* spiked frozen samples. Minced pork meat was spiked with live *Trichinella* larvae and subsequently, the spiked samples were frozen, to evaluate the possible effect of freezing on the recoverability of these larvae. The spiked and frozen samples were subjected to artificial digestion during 30 min according to EU 2075/2005 and subsequent detection of larvae by sequential sieving.

3. Validation of sequential sieving in relation to EU-RM. The sequential sieving method was validated by comparison of data obtained by analysis of fox forelegs using the EU-RM before freezing and data from digestion by SSM after freezing at −80 °C.

### Validation of larva detection

Stainless steel sieves, approximately 18 centimetres in diameter, with mesh size 300 μm, 63 μm and 38 μm were stacked in decreasing mesh size order. A mesh size of 300 μm was used to retain undigested particles, instead of 180 μm, which is used in the EU-RM for the detection of live *Trichinella* ML. This reduced the risk of losing dead, comma shaped ML, which have typical measurements of 745-975 μm length by a width of 36 μm [25]. To validate the efficacy of the smaller mesh size sieves to retain *Trichinella* larvae, 1-39 live naked ML in tap water (BIOR, Latvia) or 1-134 dead naked ML in *Trichinella*-free fox meat digest (RIVM, Netherlands) were poured into the upper, larger mesh size sieve. Subsequently, the ML were carefully washed off the sieves with tap water using a laboratory squeeze bottle, under an angle of approximately 45 degrees. ML that concentrated in the lower rim of the sieves after washing were collected in Petri dishes in approximately 20 mL of rinse water. The number of larvae per sieve was determined microscopically. The experiments were conducted by two researchers per location (BIOR and RIVM), the first author being one of them on both locations.

This sequential sieving method to isolate dead Trichinella larvae was compared to sedimentation as used in EU-RM. For this purpose, Trichinella-free meat digest was spiked with ten dead, 6-shape to comma-shaped *Trichinella* larvae, which were picked randomly and transferred to approximately 2 mL tap water. Subsequently, the larvae were rinsed into 2 liter of meat digest fluid, in twenty replicate tests. The spiked fluid was either transferred to a separatory funnel and left to sediment for 30 min, after which the lower 40 mL were sedimented again for 10 min in a glass cylinder according to EU-RM, or passed through a stack of stainless steel sieves according to SSM. Residual fluids from EU-RM were passed through a 38 μm mesh size sieve, to isolate ML that did not sediment within the given time.

### Feasibility of *Trichinella* spiked frozen samples

Six minced pork samples (100 g) were spiked with 10 live naked *T. spiralis* ML (RIVM strain, ISS14) and were frozen for two weeks at −80 °C. Three control samples spiked with 10 *Trichinella* ML were kept at +4 °C.

### Validation of sequential sieving method

To evaluate possible loss of *Trichinella* ML by freezing fox carcasses, the number of *Trichinella* ML was determined in unfrozen muscle samples of individual fox upper forelegs, originating from 35 foxes collected in Latvia as described above. Briefly, 15 gram of muscle tissue per fox leg was digested according to the EU-RM, with adaptation of the digestion fluid volume to 250 mL and the use of a 1-litre separation funnel to sediment possibly present live *Trichinella* larvae.

Thirty-five *Trichinella* positive forelegs (9 - 169 ML per 15 g muscle tissue) were frozen and kept at −80 °C for one to two weeks. Following this period, deep frozen fox legs were thawed at approximately 18 °C and kept at 8 °C until analysis within 24 h and artificial digestion was performed as described above, during 40 min, to guarantee complete matrix digestion. Liberated, naked 6-shaped to comma-shaped *Trichinella* ML were isolated by sequential sieving through a stack of 300, 63 and 38 μm mesh size sieves.

### *Trichinella* monitoring in the Netherlands

Fox carcasses were thawed at approximately 10 °C. Per individual fox, 15 g lower foreleg muscle tissue sample was isolated and pools of 4-7 foxes were digested for 40 min in 2 litre tap water of 46 °C, containing 0.5% (w/v) pepsin and 0.2% HCl (v/v) according to the EU-RM. After artificial digestion, sequential sieving through stacked stainless steel sieves with mesh size 300 μm and 63 μm was used, to isolate naked *Trichinella* ML. Foxes of pools that tested positive for *Trichinella* were re-tested individually using the same method.

### Statistical analysis

#### Validation of larva detection

*Trichinella* ML recovery data of liquid samples that were spiked with either live or dead free ML are assumed randomly distributed. Therefore, a generalized linear model approach with Poisson link function was used to fit data with and without the factor “live/dead”. Subsequently, both models were compared by likelihood ratio test to select the model with the lowest AIC-value (Akaike’s Information Criterion).

The ability of EU-RM and SSM to recover dead *Trichinella* ML from spiked meat digest was compared with Fisher’s Exact test.

#### Validation of sequential sieving method

Isolated *Trichinella* ML were counted independently by two researchers and for each fox, the average value of these two counts was used. The data were plotted and outliers were identified using Grubb’s analysis of residuals for best linear fit. Identified outliers were excluded from further analysis. Average parasite numbers before and after freezing were analysed by generalized linear model approach, with negative binomial link function. This distribution allows for overdispersion, and is therefore suitable for parasite count data that typically have a contagious distribution in host tissues [26]. We checked the prerequisite of equal variances by means of the non-parametric Bartlett test of homogeneity of variances [27]. We built a model with variate “count”, dependent on covariate “freezing status” with levels “frozen” or “fresh”. A *p*-value below 0.05 for this covariate indicates a significant effect of freezing. Statistical analyses were performed using the software package “R”, version 3.0.1 [28].

### Study in a low-endemic area in the Netherlands

Lower foreleg muscles of 369 Dutch foxes were examined in pools of 4-7 animals using artificial digestion and sequential sieving through 300 and 63 μm. One single *Trichinella* sp. ML was recovered, which was stored in 5 μL sterile DNAse free water and kept at −20 °C until further use.

### DNA isolation and molecular confirmation of *Trichinella* ML by Multiplex PCR

DNA was isolated using QIAGEN® QIAamp DNA Mini Kit Tissue Protocol. Of thirty foxes, a pool of five *Trichinella* ML was analysed per animal before freezing. The concentrations of extracted DNA in samples were measured with ND-1000 Spectrophotometer (NanoDrop Technologies, Inc., Wilmington, DE 19810, USA). The Multiplex PCR was directed at the ITS1, ITS2 and ESV genes as described by Zarlenga et al. [29]. PCR reactions were performed in a total volume of 30 μL, containing 15 μL 2× Master mix (PROMEGA M7505, USA), 1 μL of 10 pmol/μL oligonucleotide mixture, 4 μL of RNAse-free water and 10 μL of DNA. As positive control, *T. spiralis*, *T. britovi* and *T. nativa* DNA was used. The PCR conditions were 95 °C for 4 min followed by 35 cycles of 95 °C for 10 s, 55 °C for 30 s, 72 °C for 30 s. PCR products were analysed by QIAxcel ScreenGel 1.1.0 (Qiagen, Hilden, Germany) and identified according to banding pattern as described earlier [23,29]. This work was performed at BIOR (Riga, Latvia).

### DNA isolation and molecular confirmation of *Trichinella* ML by single larvae PCR

DNA was isolated from 3-4 individual ML per Latvian red fox before and after freezing, from three individual larvae from the Latvian wild boar and from the single isolated larva from Dutch red fox according to the protocol described by Pozio et al. [30]. Briefly, 2 μL of 0.05 M TRIS-HCL pH 7.6 was added to each larva in 5 μL H2O, which was overlaid with mineral oil and heated to 90 °C for 10 min. Subsequently, 0.4 μL proteinase K and 2.6 μL H2O was added, followed by incubation at 48 °C for 3 h and finally a 10 min proteinase K inactivation step at 90 °C. A single larvae PCR directed at the 5S ribosomal rDNA intergenic region was used as described earlier [31,32], to determine the species of isolated *Trichinella* ML by DNA sequence analysis, to investigate possible occurrence of simultaneous mixed *Trichinella* infections and to evaluate the influence of freezing on DNA sequencing efficacy. 5S PCR test sensitivity was determined by PCR and agarose gel analysis of four repetitive dilution series with a range of 5 ng to 1 pg *T. britovi* control DNA. PCR amplicons were purified using standard procedures (ExoSAP-IT®, Affymetrix, Cleveland, Ohio, USA). Sequence PCR reactions were carried out on both DNA strands in 20 μL final volume containing 3 μL of amplicate, 7 μL sequence buffer, 1 μL of Big Dye Terminator and 1 μL of forward or reverse PCR primer. Sequence PCR was performed under the following conditions: 95 °C for 1 min, followed by 25 cycles of 96 °C for 10 min, 50 °C for 5 min and finally 60 °C for 4 min. Trace files of the obtained sequences were generated on an automated ABI sequencer. DNA sequences were assembled, edited manually, and analysed with BioNumerics version 7.1 (Applied Maths NV, Sint-Martens-Latem, Belgium). Cluster analysis of the sequences was conducted using BioNumerics 7.1 with Jukes-Kantor correction setting and bootstrap analysis of 2500 replicates. Sequence homology ≥ 99% was considered proof of identity between isolates and available 5S rDNA sequences of *Trichinella* species from Genbank. This work was performed at RIVM (Bilthoven, Netherlands).

## Results

### Validation of larva detection

The sensitivity to detect dead *Trichinella* ML in meat digest of the EU-RM was 60% (*n* = 100), whereas the SSM performed significantly better with 92% (*n* = 100) sensitivity (*p* = 6 · 10^-12^, Fisher’s Exact test) (Table 1).

**Table 1:** **Recovery of dead** ***Trichinella*** **larvae spiked in meat digest**

		**EU 2075 2005**		**SSM**	
	**Spike**	**Sedimentation**	**Residual fluids** ^*^	**63 μm**	**38 μm**
**1**	10	4	6	8	0
**2**	10	7	3	8	1
**3**	10	2	8	9	0
**4**	10	4	5	10	0
**5**	10	6	3	10	0
**6**	10	9	1	10	0
**7**	10	6	3	8	0
**8**	10	8	2	10	0
**9**	10	8	2	10	0
**10**	10	6	4	9	0
sum:	100	60	37	92	1

Ten dead, 6-shape to comma-shaped *Trichinella* larvae were picked randomly and transferred to approximately 2 mL tap water. Subsequently, the larvae were rinsed into 2 liter of meat digest fluid. The spiked fluid was either transferred to a separatory funnel and left to sediment for 30 min according to EU 2075/2005, or passed through a stack of stainless steel sieves according to SSM. SSM performed significantly better than EU-RM for detection of dead larvae in meat digest (*p* =6 · 10^-12^, Fisher’s Exact test).

^*^ # of larvae found after sieving the residual fluids through 38 μm sieve following sedimentation.

**Figure 2:**
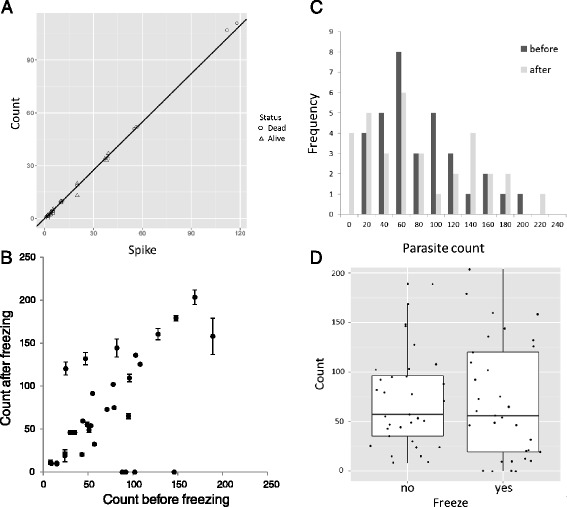
**Recovery of dead or live** ***Trichinella*** **larvae. A**. Fourty-one data points of two combined experiments using the SSM are shown: single to fourfold spikes and counts of dead larva (20 samples, RIVM) and triplicate spikes and counts of live ML (21 samples, BIOR). Identical data points from the same experiment appear as one single data point in the graph. **B**. *Trichinella* larvae were isolated using the EU-RM for live larvae (before freezing) and by the SSM for dead larvae (after freezing). Individual data points represent average values of duplicate counts by two researchers; error bars represent counts range. One identified outlier is omitted here. **C**. Parasite counts mentioned under **A** display a negative binomial distribution. **D**. Parasite counts before freezing (freeze no) and after freezing (freeze yes) overlap and median values before (57) and after (56) freezing were comparable. Top and bottom of the boxes represent 25^th^ and 75^th^ percentiles respectively.

Overall sensitivity of the sequential sieving to detect *Trichinella* ML was 92.9% when using dead ML (*n* = 451) and 88.9% (*n* = 280) for samples spiked with live ML. Using the recovery data of the spiked samples, a Poisson generalized linear model was fitted with and without the factor “live/dead”. Comparing both models, the model without “live/dead” factor was favoured resulting from lower AIC-value (Akaike’s Information Criterion) and a *p*-value of 0.58 after likelihood ratio testing. The best fitting model to describe the relationship between the number of spiked and counted larvae was *count* =0.91*spike*, the slope of which is close to, but significantly different from 1 (*p* = 0.0198) (Figure 2A).

In total 2833 dead *Trichinella* ML were isolated from 31 frozen Latvian fox forelegs by sequential sieving, of which 0.4% (12 ML) passed through the 63 μm mesh size and were retrieved from the 38 μm mesh size sieve. Of live larvae, 5.8% (14 out of 243) passed through the 63 μm sieve and were collected from the underlying 38 μm sieve. From these results, it was decided to use a combination of sieves with mesh size 300 μm and 63 μm to study *Trichinella* prevalence in deep-frozen foxes from a low-endemic area (the Netherlands).

### Effect of freezing on *Trichinella* larvae

Minced pork samples were spiked with free larvae (without nurse cell), to increase the precision of recovery evaluation. Detection of *T. spiralis* (RIVM strain, ISS14) ML in frozen pork samples spiked with 10 ML using artificial digestion according to EU-2075/2005 with 30 min digestion time and subsequent detection of larvae by sequential sieving, showed a sensitivity of only 48.3% (*n* =60), whereas the recovery from control samples stored at +4 °C was 80% (*n* =30) (data not shown). It was then decided to abandon this artificial line of evaluation and to continue the validation with naturally infected fox forelegs before and after freezing, since the latter was to be used for the prevalence study in a low-endemic area.

### Validation of sequential sieving method

Given the poor performance of EU-RM to detect dead Trichinella ML in meat digest, and the fact that about 6% of live ML actively pass the 63 μm sieve with SSM, it was decided to compare the most efficient method to detect live *Trichinella* ML in non-frozen meat (EU-RM) with the best method to detect dead *Trichinella* ML in frozen meat (SSM). In most cases, parasite counts in 35 Latvian fox forelegs before and after freezing were comparable (Figure 2B); in one occasion 575 *T. britovi* ML were found after freezing, against 150 prior to freezing (data not shown). This count was identified as a significant outlier in Grubb’s test and therefore excluded from further analysis (G =4.5713, U =0.3476, *p* =1.3 · 10^-7^). In four samples no ML were found after freezing against 88-146 ML before freezing, which might be related to the highly uneven distribution of *Trichinella* in host muscle tissue and the dispersed count data. Indeed, parasite counts showed a skewed frequency distribution consistent with a negative binomial distribution (Figure 2C). This was confirmed by testing these data for overdispersion (Z =6.5193, *p* =3.5 · 10^-11^) [28]. Median parasite counts of the fox legs before and after freezing were highly similar with 57 and 56 ML respectively (Figure 2D). Variances were not significantly different (K-squared =1.6677, df =1, *p* =0.1966, non-parametric Bartlett test of homogeneity of variances) and GLM analysis of parasite counts with the variable “freeze” as factor revealed no significant difference (Z = -0.068, *p* =0.946).

### Study in a low-endemic area in the Netherlands

**Figure 3:**
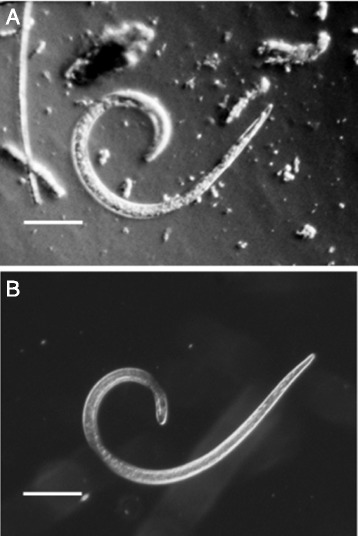
**Single** ***Trichinella*** **larva isolated from Dutch red fox. A**. One larva was isolated from a fox carcass that had been frozen at −80 °C for one week. Note the retracted granular inner structure of the larva. No PCR product could be generated from this specimen. **B**. Dead (unfrozen) comma shaped *T. spiralis* larva. Original magnification 46×, Olympus BH-2 microscope, maximum contrast settings), bars represent 100 μm.

One fox out of 369 tested positive for *Trichinella*, with one larva (Figure 3) found in a pool of six foxes. Analysis of the individual foxes that were included in the positive pool did not lead to further findings. Assuming constant prevalence over the study period, we may combine all study years, to arrive at a prevalence of 0.27% (95% CI 0.065-1.5%). Prevalence calculated only from the 287 foxes collected from November 2010 - April 2011 reached 0.35%. In contrast, analysis of 276 foxes from a previous study at the eastern border region of the Netherlands (the same region as in this present study), collected from December 1997 - March 1998 [8], revealed a significantly higher *T. britovi* prevalence of 3.9% (*p* =0.0006, Fisher’s Exact Test) at a density of 0.04 - 0.71 LPG [8]. Also in the period 1969 - 1971, a significantly higher prevalence (2.8%, *n* =106) compared to this present study, was found in foxes from the same border region by digestion and subsequent sieving through sterile gauze [5], (*p* =0.036, Fisher’s Exact Test).

### Molecular characterization of *Trichinella* ML

#### Multiplex PCR

Multiplex PCR on five isolated ML each of 30 individual Latvian foxes at BIOR (Riga, Latvia) showed that 28 animals were infected with *T. britovi* and two with *T. nativa*. It is not possible however, to detect simultaneous *T. britovi* and *T. nativa* infections by multiplex PCR, since banding patterns on gel do overlap (one single band of 127 base pairs (bp) for *T. nativa* and 2 bands of 253 and 127 bp respectively for *T. britovi*).

#### Single larva PCR

Single larva PCR directed at 5S rDNA on three to four individual non-frozen ML per Latvian fox performed at RIVM (Bilthoven, the Netherlands), confirmed the results of multiplex PCR performed at BIOR, without any mixed *T. britovi* and *T. nativa* infection found. Of in total 96 tested non-frozen *T. britovi* ML, 90 (93.8%) were successfully sequenced, whereas only 6 out of 72 (8.3%) frozen *T. britovi* ML yielded sequences that allowed species determination (Table 2). For the more freeze-resistant *T. nativa*, six out of six non-frozen and two out of six frozen ML were successfully sequenced. The detection limit of the 5S rDNA PCR was 2.5 pg (data not shown).

**Table 2:** **Species identification of Trichinella larvae**

**#**	**Animal**	**Multiplex PCR before freezing**	**Single larva 5S PCR before freezing**				**Single larva 5S PCR after freezing**		
**1**	67038	*T. britovi*	*T. britovi*	*T. britovi*	*T. britovi*	*T. britovi*	NP	NP	NP
**2**	70414	*T. britovi*	*T. britovi*	*T. britovi*	*T. britovi*	*T. britovi*	NP	NP	NS
**3**	72119	*T. britovi*	*T. britovi*	*T. britovi*	*T. britovi*	*T. britovi*	NP	NP	NP
**4**	72407	*T. britovi*	*T. britovi*	*T. britovi*	*T. britovi*	*T. britovi*	NP	NP	NP
**5**	74391	*T. britovi*	*T. britovi*	*T. britovi*	NP	NP	NP	NS	NP
**6**	75633	*T. britovi*	*T. britovi*	*T. britovi*	*T. britovi*	*T. britovi*	NP	NP	NP
**7**	75068	*T. britovi*	*T. britovi*	*T. britovi*	*T. britovi*	*T. britovi*	NP	*T. britovi*	*T. britovi*
**8**	74497	*T. britovi*	*T. britovi*	*T. britovi*	*T. britovi*	*T. britovi*	NP	NP	NP
**9**	75475	*T. britovi*	*T. britovi*	*T. britovi*	*T. britovi*	*T. britovi*	*T. britovi*	NS	NS
**10**	75748	*T. nativa*	*T. nativa*	*T. nativa*	*T. nativa*	ND	NP	NP	NP
**11**	75630	*T. britovi*	*T. britovi*	*T. britovi*	*T. britovi*	ND	NP	*T. britovi*	NP
**12**	75638	*T. britovi*	*T. britovi*	*T. britovi*	*T. britovi*	ND	NP	NP	NP
**13**	75932	*T. britovi*	NP	*T. britovi*	*T. britovi*	ND	NP	NP	NP
**14**	75933	*T. britovi*	NP	*T. britovi*	*T. britovi*	ND	NP	NP	NP
**15**	75996	*T. britovi*	*T. britovi*	*T. britovi*	*T. britovi*	ND	*T. britovi*	*T. britovi*	NS
**16**	76148	*T. nativa*	*T. nativa*	*T. nativa*	*T. nativa*	ND	NP	*T. nativa*	*T. nativa*
**17**	76575	*T. britovi*	*T. britovi*	*T. britovi*	*T. britovi*	ND	NP	NP	NP
**18**	76580	*T. britovi*	*T. britovi*	*T. britovi*	*T. britovi*	ND	NP	NP	NP
**19**	76643	*T. britovi*	*T. britovi*	*T. britovi*	*T. britovi*	ND	NP	NP	NP
**20**	76644	*T. britovi*	*T. britovi*	NP	NP	ND	NP	NP	NP
**21**	76806	*T. britovi*	*T. britovi*	*T. britovi*	*T. britovi*	ND	NP	NP	NP
**22**	77876	*T. britovi*	*T. britovi*	*T. britovi*	*T. britovi*	ND	NP	NP	NP
**23**	77885	*T. britovi*	*T. britovi*	*T. britovi*	*T. britovi*	ND	NP	NP	NP
**24**	77958	*T. britovi*	*T. britovi*	*T. britovi*	*T. britovi*	ND	NP	NP	NP
**25**	78187	*T. britovi*	*T. britovi*	*T. britovi*	*T. britovi*	ND	ND	ND	ND
**26**	71102	*T. britovi*	*T. britovi*	*T. britovi*	*T. britovi*	ND	ND	ND	ND
**27**	71127	*T. britovi*	*T. britovi*	*T. britovi*	*T. britovi*	ND	ND	ND	ND
**28**	71128	*T. britovi*	*T. britovi*	*T. britovi*	*T. britovi*	ND	ND	ND	ND
**29**	74449	*T. britovi*	*T. britovi*	*T. britovi*	*T. britovi*	ND	ND	ND	ND
**30**	74956	*T. britovi*	*T. britovi*	*T. britovi*	*T. britovi*	ND	ND	ND	ND
**31**	wild boar	ND	*T. britovi*	*T. britovi*	*T. britovi*	ND	ND	ND	ND

Species identification was performed on pools of 5 larvae (multiplex PCR) and individual *Trichinella* larvae (single larva PCR). PCR on individual non-frozen larvae resulted in product for 93 out of 99 larvae (93.9%). PCR on 72 individual frozen larvae yielded PCR product for only 12 larvae, of which 8 resulted in sequence product. NP: no PCR product was formed. NS: PCR product yielded no sequence results due to poor quality of DNA. ND: not done.

The single microscopically identified *Trichinella* sp. larva that was recovered from 369 frozen lower forelegs of Dutch foxes appeared severely damaged (Figure 3) and did not result in PCR product after 5S PCR and therefore, no sequence was available for species determination of this isolate.

## Discussion

A method using sequential sieving (SSM) for the detection of dead *Trichinella* ML from frozen red fox foreleg muscle was validated and was used to analyse trends in time of *Trichinella* in a Dutch red fox population. The SSM is a fast method, since two sedimentation steps of minimally 30 min primary sedimentation plus 10 min secondary sedimentation (when using the EU-RM) were eliminated and were replaced by a 3-5 min sieving step in the SSM.

Dead Trichinella ML exhibit a lower sedimentation speed than live ML [4,19,20] leading to only 60% recovery of dead Trichinella ML from muscle digest using EU-RM, compared to 92% when using SSM, as is shown in this present paper. In comparison, larval counts of frozen fox foreleg muscle obtained with SSM did not differ significantly from larval counts of non-frozen fox foreleg muscle obtained with EU-RM, showing that the SSM was effective to detect dead *Trichinella* larvae. Finding or preparing suitable samples for this type of comparison is a challenge. Henriksen [21] used minced and thoroughly mixed experimentally infected rabbit meat to evaluate the effect of freezing on recoverability of *T. spiralis* ML using disposable sieves with mesh size 350 and 20 μm to retain dead larvae. Parasite counts ranged from 82 to 124 ML in that study, irrespective of temperature treatment, despite thorough mixing. In our validation experiment, we found four negative counts after freezing of samples that contained 82-146 larvae when tested before freezing. A plausible biological explanation could be that due to uneven distribution of *Trichinella* larvae in the muscle tissue, these could be missed by chance at second sampling of the same foreleg, near to the primary sampling site. This could also explain the same effect in the other direction, where the post-freezing count value of one sample was 383% of the pre-freezing count. The statistical analysis on the parasite counts in this present study confirmed that parasites follow a contagious distribution in tissues, necessitating GLM methods to accommodate such highly variable counts.

Detection of live and dead *Trichinella* larvae using sequential sieving showed an average sensitivity of 91% (*n* =451). Spiked samples with live naked or encapsulated *T. spiralis* ML provide standardized, uniform and quantifiable samples to evaluate test sensitivity of the EU-RM in routine laboratories. This type of samples are generally used by all National Reference Laboratories for *Trichinella* in Europe and elsewhere, with a quantitative sensitivity of 84% (*n* =2130, naked larvae) [33] and 81% - 88% (*n* =174 - 265, encapsulated larvae) [34] under controlled circumstances. This method however, seems less suitable to validate the SSM presented in this paper, since the test sensitivity dropped from almost 93% (validation of mesh size) to 48% (SSM, *n* =60) after freezing of pork samples, that had been spiked with live *T. spiralis* ML, for two weeks (at −80 °C). Test sensitivity of unfrozen spiked control samples that were stored at +4 °C was 80% (*n* =30). This low recovery after freezing was confirmed by a study of Nga [35], who analysed pork samples that were spiked with live *T. spiralis* ML (the same strain as was used in this present study) and were subsequently frozen at −20 °C for at least three weeks. Using the EU-RM, the test sensitivity of *Trichinella* detection was 56% (*n* =225) after freezing in that study, whereas the test sensitivity was 91% (*n* =225) for control samples that had been stored at +4 °C [35]. Dead ML were found only occasionally, indicating destruction of *T. spiralis* ML during freezing. In an earlier study, Jackson [36], demonstrated even 78% loss of *T. spiralis* larvae (compared to non-frozen samples) after freezing at −18 °C. Also in that study, dead larvae were found occasionally.

The use of free larvae without a nurse cell both in the present study and in that of Nga [35] alone, could not explain the large drop in larval recovery after freezing, since Randazzo et al. [17] found no protective effect of the nurse cell capsule against low temperature treatment. An explanation for the lower results with *T. spiralis* spiked frozen samples, could be difference in freeze tolerance between *T. spiralis* and *T. britovi* muscle larvae. Lacour et al. [18] indeed found a *T. spiralis* ML inactivation half time of 25 h at −21 °C, whereas 35 h at −21 °C were needed to inactivate half of *T. britovi* ML. However, after one week at −18 to −30 °C, both *T. spiralis* and *T. britovi* that were recovered from either experimentally infected wild boar, rat or mouse muscle tissue, were unable to infect mice [15,16,18,37]. In naturally infected carnivore muscles, the survival time of *T. britovi* at −15 to −20 °C is considerably longer, with 3-6 months, but this trait is lost with the transfer of the parasite to experimental mice [15]. This effect might also have induced the dramatic decline in *T. spiralis* recovery after freezing in our spike experiment and that of Nga [35]. The *T. spiralis* strain that Jackson used for his freezing experiment mentioned above, was maintained for almost 40 years [36]. More importantly, these observations underscore our preference for naturally infected fox legs to validate the SSM.

In summary, we validated a fast and effective method to detect dead larvae in meat samples of wildlife. Using this method, we analysed 369 Dutch foxes, of which only one pool of six foxes was positive for *Trichinella.* In this pool, one single larva was isolated and re-tested samples of individual foxes belonging to this pool were all negative, showing a very low infection level.

The *Trichinella* prevalence found in this present study was ten times lower than that described in 1972 by Sluiters et al. [5] and in 1998 by Van der Giessen et al. [8]. Detailed literature concerning historical data regarding *Trichinella* prevalence in red fox from adjacent areas is scarse. However, in the bordering north-western part of Germany (state Hessen), the prevalence of Trichinella in red foxes in the period 1980 - 1983 was 3% (trichinoscopy, six positive, *n* =198), whereas in the preceding (1979 - 1980) and following period (1985 - 1987) no positive foxes were found there using artificial digestion (*n* =410 and 333 respectively) [38]. In Nordrhein-Westfalen, situated in-between Hessen and the Netherlands, *Trichinella* was reported in badger (*Meles meles*, 1985) and in wild boar (1988), however no prevalences were given [38]. During the hunting season of 2012, in the eastern part of Belgium (Flanders), one *Trichinella* sp. larva was found in a pool of 20 foxes and also in this occasion, it was not possible to identify an individual positive fox [13], whereas Geerts et al. [39] were not able to demonstrate *Trichinella* in 116 Belgian red foxes in 1993. The decline in *Trichinella* (*britovi*) prevalence in the Netherlands over the past 15 years fits the prevalence patterns of surrounding countries and might be driven by changing feeding habits of the opportunistic red fox in an increasingly densely populated area as the Netherlands. However, not much is known about the natural prevalence fluctuation or infection dynamics of *T. britovi* in red fox. In Slovakia, in contrast to the situation in the Netherlands, the prevalence of *Trichinella* spp. in red fox increased fourfold during the period 2000 - 2007 [12].

Efforts to identify the species of the single larva found in Dutch foxes by PCR failed, probably due to severe freezing damage, which was clearly visible microscopically. Using validation samples from naturally infected Latvian foxes, we were able to determine a success rate of only 8.3% (*n* =72) for molecular speciation of frozen *T. britovi* ML by 5S PCR, against 94% (*n* =96) for live larvae prior to freezing. The purpose of testing frozen larvae in our setting was to determine the probability of obtaining positive identification using the 5S PCR on individual larvae that had been submitted to freezing at −80 °C for at least one week, since this information was not available in literature up to date.

Several studies report species identification of field samples that were frozen at −20 °C, using single larva multiplex PCR [10,40-43]. None of these studies however, stated the number of single larvae tested per host animal, or the success rate. One study by Pozio et al. [30] on wildlife samples frozen at −20 and −80 °C, used 5 to 10-fold single larva multiplex PCRs to identify the Trichinella species, but did not mention how many of these larvae actually were identified. The use of multiple attempts in that study implicated that it was at least anticipated to have a low success rate. Moreover, in a study in coyotes with very low *Trichinella* intensity (0.05-0.6 LPG) [44], Trichinella species identification was possible using multiplex PCR in 7 out of 9 animals after freezing of the samples at −20 °C.

The 5S PCR method displayed a test sensitivity of 2.5 pg larval DNA in our laboratory. This level is in range with a sensitivity of 1 pg DNA in a conventional PCR targeted at mitochondrial large subunit RNA of *T. spiralis* as demonstrated by Lin et al. [45]. Other methods like Q-PCR and multiplex PCR may be more sensitive than the 5S PCR, to identify sheared and otherwise damaged larval DNA after freezing, since these PCR methods usually target much smaller DNA fragments. To increase species identification sensitivity, a combination of methods may be considered.

Molecular identification of individual *Trichinella* larvae revealed two species in red fox from Latvia: *T. britovi* and *T. nativa*, without any mixed infection in 30 foxes. Malakauskas et al. [10] demonstrated *Trichinella* spp. prevalence of 29% in foxes in Latvia. In that publication, individual larvae were identified with PCR according to Pozio et al. [30], which showed a distribution of 78% *T. britovi*, 8.5% *T. nativa* and 9.3% mixed infection of the two species in 129 Latvian foxes. Although our sample size of Latvian foxes is much lower and primarily aimed at the validation of our method, we found a comparable distribution of *T. britovi* and *T. nativa*. The number of isolated *Trichinella* ML from Latvian foxes in this present study might be too low to demonstrate mixed infections.

In conclusion, this study presents a fast and effective sequential sieving method for the detection of dead *Trichinella* larvae in frozen meat. Using this method, we showed that in contrast with a study in the same area fifteen years ago using a comparable method, *Trichinella* prevalence in a Dutch red fox population was significantly lower. Moreover, this study demonstrated that the efficacy of 5S PCR for identification of *Trichinella britovi* single larvae from meat that had been deep-frozen is not more than 8.3%. This is the first time that the effect of deep freezing on *Trichinella* species identification was quantified. To increase species identification sensitivity and at the same time generate DNA sequence information for molecular epidemiology, a combination of methods may be considered.
